# A study on the spatial-temporal patterns and influencing factors of atmospheric vulnerability in the Pearl River Delta

**DOI:** 10.1371/journal.pone.0289436

**Published:** 2023-11-09

**Authors:** Bo Tang, Zhixiong Tan

**Affiliations:** Guangzhou Xinhua University, School of Resources and Planning, Guangzhou, China; Guangzhou Institute of Geography, Guangdong Academy of Sciences, CHINA

## Abstract

Atmospheric environmental assessment has emerged as a prominent area of research due to global climate change and regional atmospheric pollution issues. Accurate evaluation of atmospheric environmental vulnerability characteristics and understanding driving mechanisms are crucial for effective air pollution monitoring and prevention. This study focuses on the Pearl River Delta (PRD) region and employs the Vulnerability-Scoping-Diagram (VSD) model framework to establish an index system for assessing atmospheric environmental vulnerability based on exposure, sensitivity, and adaptability, combining the entropy value method and adopts Geographic Information System (GIS) for the time change and spatial evolution analysis, and finally utilizing the factor detection and interaction in Geodetector to explore the contribution degree of each driving factor of atmospheric environmental vulnerability and the exchange of influencing factors. The findings of this research are as follows: Firstly, the sensitivity index and resilience index of the atmospheric environment of the PRD exhibit an overall upward trend with fluctuations, while the exposure index demonstrates a pattern of initial increase, followed by a decrease, and subsequent increase with significant interannual variability. Secondly, the atmospheric environment vulnerability level of the PRD is primarily categorized as low and mild, with a negligible proportion of moderate vulnerability and no instances of severe or extreme vulnerability. The vulnerability index shows an initial increase followed by a subsequent decline from 2016 to 2020, indicating an overall improvement in the region’s atmospheric environment. Thirdly, notable variations exist in the atmospheric environment vulnerability indices among the nine cities in the PRD, in which moderate vulnerability and low vulnerability are mainly concentrated in Guangzhou, Shenzhen, Foshan, and Dongguan in the central part of the PRD. lower vulnerability is primarily observed in the eastern and western regions of the PRD characterized by favorable natural environments and limited human interference, such as Huizhou, Zhaoqing, and Zhuhai. Finally, the atmospheric environment vulnerability of the PRD is the result of the combined effect of various driving factors, among which the urban built-up area, PM2.5 concentration, SO2 concentration, population density and the share of tertiary industry in GDP are the key drivers.

## Introduction

With the increasing concern over global climate change, the state of the atmosphere has garnered significant attention from governments and the general public. In recent years, China’s rapid socioeconomic development, population increase, and urban expansion have resulted in the substantial emission of harmful gases during human activities, exacerbating atmospheric pollution and posing serious threats to the ecological environment and climate change. Therefore, in response to the air pollution problem, to better carry out the prevention of air pollution and environmental protection, both the central and local governments have implemented a series of laws and policies aimed at controlling air pollutant emissions. Atmospheric pollution occurs when some pollutants enter the atmosphere under natural or artificial factors. Over time, these pollutants accumulate and reach a concentration level that adversely affects human health and the natural ecological environment [1]. As such, the assessment of ecological and environmental vulnerability necessitates a thorough understanding of atmospheric pollution. Presently, the phenomenon of a heavy regional atmosphere has become one of the bottlenecks limiting the sustainable socio-economic development of China, seriously threatening the living environment and health of the people [2]. How to reconcile the complex interplay between socioeconomic development and the atmospheric environmental system has become an essential part of a sustainable development strategy.

Vulnerability research originated from exploring natural hazards and has evolved into a frontier scientific issue in studying the interactions of coupled human-environment systems. The concept of vulnerability was first introduced by Timmerman in 1981, defining it as the extent to which a system reacts negatively to the occurrence of a hazardous event [3]. In the 1970s, the study of vulnerability was introduced into the field of natural hazards [4, 5] and subsequently gradually expanded to the area of humanities and social sciences, with applications in various disciplines such as geography [6], ecology [7], disaster science [8], economics [9] and sociology [10]. However, the interpretation and understanding of "vulnerability" differ among experts and scholars due to their diverse disciplinary perspectives and research domains. Downing was the first to provide a definition for "vulnerability science" in the 2000 International Human Dimensions Programme on Global Environmental Change, outlining its key characteristics and research objectives. Vulnerability is regarded as a property of a system (subsystem, system component) that is prone to structural and functional changes due to its sensitivity to internal and external disturbances and its limited coping capacity. This sensitivity is manifested through the system’s responsiveness to concerns and power dynamics, influencing its internal characteristics [11]. At the beginning of the 21st century, studies on ecological vulnerability have gradually become the focus of many scholars at home and abroad. Researchers have predominantly focused on the impact of ecological vulnerability in the context of climate change. Minnen et al. argued that global climate change would cause a trend of decreasing precipitation in southern Europe in the next 100 years, leading to a notable increase in the vulnerability of natural vegetation [12]. Similarly, Janowiak et al. examined the vulnerability of forest ecosystems in northern Wisconsin and western Upper Michigan, USA, under the influence of climate change [13]. International vulnerability studies tend to encompass broader scales, such as watershed, national and provincial perspectives [14]. For example, Sherbinin compared the differences in vulnerability among three coastal cities,—Mumbai, Rio de Janeiro, and Shanghai—in response to global climate change [15]. Geographic information systems and data models are commonly employed in vulnerability research, and five primary theoretical and assessment frameworks have emerged: risk-hazard model, pressure-release model, regional vulnerability model, dual structure model, and coupled system model. Among them, the Turner coupled system collects them, considering the degree of exposure, sensitivity, and resilience as the focus of vulnerability research [16]. For example, Adger suggested that ecological vulnerability consists of exposure, sensitivity and resilience, and is an expression of a system’s ability to cope with external disturbances and its level of recovery [17]. Smith and Pilifosova argued that the vulnerability of a given process should be linked to its ability to adapt to the effects of climate change, with vulnerability being a function of exposure and adaptive capacity [18]. On the other hand, Ippolito proposed a research methodology that applies an ecosystem vulnerability index from three perspectives—sensitivity, exposure, and recovery potential for vulnerability assessment of two river ecosystems in northern Italy [19]. The management of ecological vulnerability is also receiving increasing attention, with exploration of both top-down and bottom-up management models and an emphasis on regional cooperation. These trends reflect the multidisciplinary, regional, and integrated nature of contemporary ecological vulnerability studies.

Vulnerability research in China has lagged behind that of foreign countries. In the 1980s, it mainly focused on studying climate change and ecological vulnerability research [20] gradually expanding to economics, sociology, and sustainable development. In recent years, the rapid growth of China’s economy and society, coupled with extensive urban construction, mineral extraction, deforestation, and other human activities, has further exacerbated the conflict between human actions and the natural environment [21]. At the same time, with the promotion of the concept of ecological civilization, the study of environmental vulnerability has gradually gained the attention [22]. In terms of research methods, various models have been developed for constructing environmental vulnerability evaluation indices, including Pressure-State-Response (PSR) [23], Vulnerability-Scoping-Diagram (VSD) [24], "Driver-Pressure-State-Impact-Response (DPSIR)" [25], and "Multi-System Integrated Evaluation" etc. The evaluation methods primarily involve the application of fuzzy comprehensive evaluation method, entropy value method, hierarchical analysis method, principal component analysis method, and analysis method based on remote sensing and GIS, etc. [26, 27]. For instance, He et al. utilized spatial principal component analysis and hierarchical analysis to construct an evaluation index system to comprehensively evaluate the vulnerability of the Yangtze River estuary marine ecosystem [28]; Zhang et al. applied the SRP model to explore the ecological vulnerability of the southwest Guizhou region, adopting a mountain-river-sea perspective [29]. Liu et al. employed the fuzzy comprehensive evaluation method to evaluate and categorize the vulnerability of water resources in ten prefecture-level cities in Shanxi Province on the structure and function of the water resources system [30]. These studies have laid the foundation for the protection of natural resources and the development of ecological zoning in China.

The concept of atmospheric environmental vulnerability has emerged from studies in ecological and environmental vulnerability due to the interdisciplinary nature of vulnerability research. It mainly refers to the sensitive response and self-recovery capability of the atmospheric environmental system in response to external disturbances, reflecting the combined influence of the atmospheric environment and human economy and society [31]. This perspective offers new insights into the management of atmospheric environmental pollution and sustainable development. The concept of vulnerability of the atmospheric environment is multifaceted, and it is the result of the combination of various factors, such as natural system stress and human society, making it an objective, universal, dynamic, and region-specific phenomenon. Especially in recent years, in the context of global climate change, atmospheric environmental vulnerability as a new research perspective has received more attention from scholars. Zhang Yang et al. conducted a study on the impact of human activities on atmospheric environmental vulnerability using multi-criteria decision analysis. They employed ordered weighted averages and developed an atmospheric environmental vulnerability evaluation model [1]. Li et al. examined the spatial and temporal evolution of atmospheric environmental vulnerability in Liaoning Province using time-series global principal component analysis [32]. However, it is important to note that the research on atmospheric environmental vulnerability is still in the exploratory stage. Most scholars’ research on atmospheric environmental vulnerability is mainly based on single-factor analysis or independent spatial comparative analysis, with limited research on vulnerability evaluation from the perspective of the atmospheric environmental system. There is a scarcity of studies that incorporate spatial analysis, temporal dynamics, and multiple factors. In contrast, the impact mechanism of atmospheric environmental vulnerability and the coupling with urban economic development needs deeper investigation.

Changing the energy structure, improving energy efficiency and reducing the emission of greenhouse gases are not only an urgent need to address climate change but also essential actions to combat environmental pollution, protect air quality and ensure the health needs of the people [20]. As the earliest region to undergo reform and opening up in China, the PRD is one of China’s three major urban agglomerations and regions with rapid economic development and growing international influence. The intensified urbanization and socio-economic development within the PRD have led to heightened human activities, presenting substantial challenges to the ecological environment. Against the backdrop of global climate change, it is of great theoretical and practical significance to investigate the spatial and temporal distribution characteristics as well as the influencing factors of atmospheric environmental vulnerability in urban agglomerations. This research endeavor aims to address contradictions between urban development, human activities, and environmental protection, to strike a balance between economic and social development and ecological civilization construction, and to promote the future high-quality development of China’s urban agglomerations.

Climate change and environmental issues exert a profound influence on global natural ecosystems and socio-economic systems, potentially impeding the sustainable development of humanity as a whole. Therefore, research on the vulnerability of the atmospheric environment can facilitate the proper use of atmospheric resources, advance precise measures for preventing and controlling atmospheric pollution, and provide a research basis for achieving the harmonious and sustainable coexistence of the atmosphere and human economic and social systems. Drawing on the evaluation of ecological environmental vulnerability, this study adopts the VSD model and constructs an evaluation system from natural, social, and economic aspects. By analyzing the spatial and temporal evolution of the atmospheric environmental vulnerability of the PRD, employing Geodetector to explore the influencing factors of atmospheric environmental vulnerability. To analyze the atmospheric environment vulnerability of urban agglomerations, a comprehensive approach including "theory construction, evaluation system, temporal changes, spatial differences, and influencing factors" is adopted. This research contributes to new perspectives and ideas for atmospheric environmental vulnerability evaluation. What is more, it contributes to the enrichment of methodologies employed in the evaluation of atmospheric environmental vulnerability. The findings of this study will serve as a reference for air pollution management and environmental protection urban agglomerations throughout China.

## Methods

**Fig 1:**
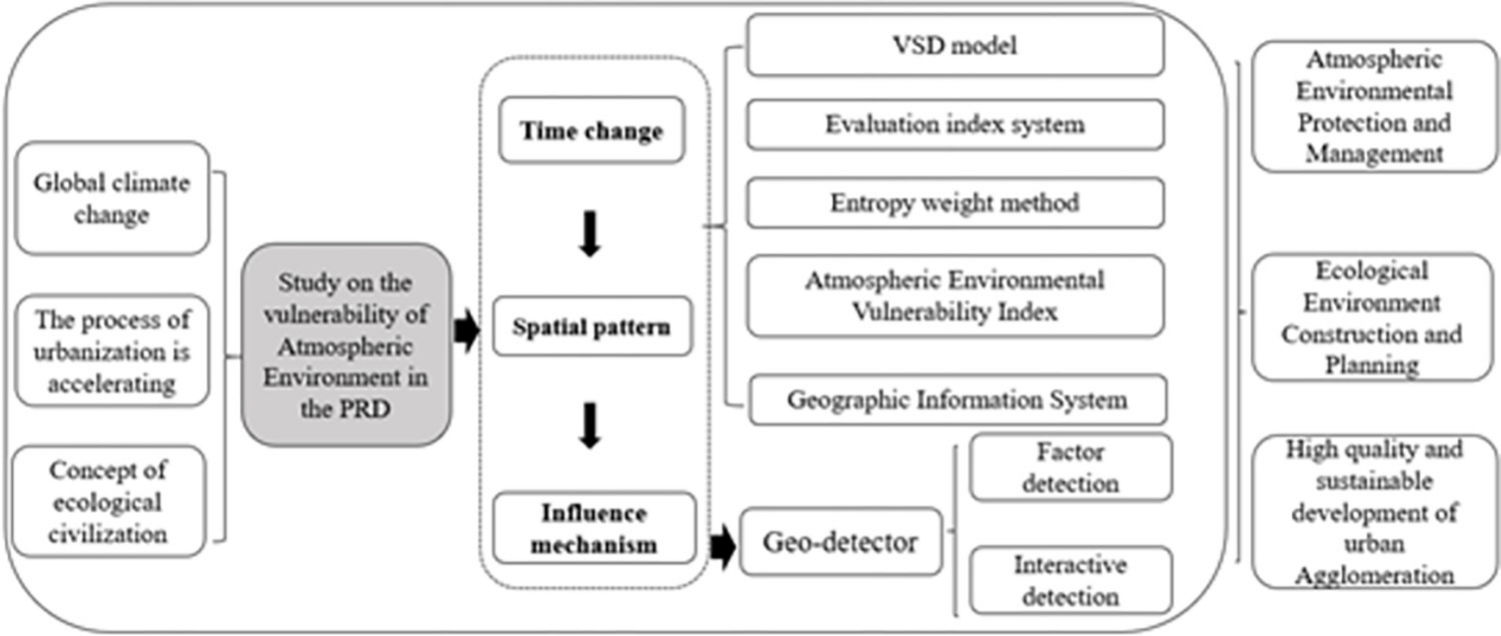
Methodological framework.

Fig 1 illustrates the methodological framework employed in this study. The VSD model is used to construct an evaluation index system for the atmospheric environmental vulnerability. Based on relevant literature, 15 evaluation indicators are selected according to the study area’s atmospheric environmental conditions and regional characteristics. The entropy value method is integrated into the analysis, for the purpose of determining the weights, calculating the exposure, sensitivity, adaptability, and vulnerability, and analysing the temporal and spatial evolution trends of atmospheric environmental vulnerability in the PRD by GIS. What is more, we’ve incorporated factor detection and interaction detection from the Geodetector methodology to delve into the main influencing factors.

### Evaluation index system construction

Vulnerability is a multidimensional concept characterized by a high degree of uncertainty in both its measurement and categorization. The VSD model was adopted to evaluate the vulnerability of the air environment in the PRD. The model integrates elements of the natural environment and human activities, mainly composed of three layers: exposure, sensitivity, and adaptability [33]. The study has taken into account the actual situation of the PRD and the accessibility of data, 15 index factors were finally selected to construct an index system for the evaluation of the atmospheric environmental vulnerability [2, 21, 31]. What is more, the significance of relevant indexes and their relationships with atmospheric environmental vulnerability were elucidated (Table 1).

**Table 1:** Evaluation index system.

Target layer	Criterion layer	Indicator number	Factor layer	Factor meaning	Indicator properties
Atmospheric Environmental Vulnerability Assessment	Exposure	*X*_1_	*SO*_2_Concentration	Reflect the level of air pollution	(+)
		*X*_2_	*NO*_2_Concentration	Reflect the level of air pollution	(+)
		*X*_3_	*PM*_2.5_Concentration	Indicates the level of respirable suspended particulate matter in the atmosphere	(+)
		*X*_4_	*PM*_10_Concentration	Indicates the level of delicate particulate matter in the atmosphere	(+)
		*X*_5_	Number of days when AQI reaches or exceeds Grade II	Indicates the number of days in which the air quality goes the standard throughout the year	(-)
	Sensitivity	*X*_6_	Annual average temperature	Indicates the average yearly temperature of the region	(+)
		*X*_7_	Average annual precipitation	Indicates the average yearly rainfall in the region	(-)
		*X*_8_	Natural population growth rate	Reflect the trend and speed of regional population change	(+)
		*X*_9_	Population density	Indicates the number of people per unit of land area, reflecting the population carrying capacity of the area	(+)
		*X*_10_	Urban built-up area	It indicates the city’s actual development and construction area and reflects the size of the urbanization area.	(+)
		*X*_11_	Energy consumption per GDP	Reflect the quality of regional economic development	(+)
	Adaptive capacity	*X*_12_	Proportion of tertiary industry in GDP	Reflect the industrial structure of regional economy and the level of economic development	(-)
		*X*_13_	Proportion of science and technology investment in GDP	Reflect regional financial support for scientific and technological research and development activities	(-)
		*X*_14_	Public green area per capita	An important index reflecting the living environment and quality of urban residents	(-)
		*X*_15_	Forest coverage	Represents forest area as a percentage of total land area	(-)

Exposure assessment is a key part of vulnerability assessment, which refers how much an individual, group, or system is affected by specific pressures or shocks [34]. Atmospheric pollution represents one of the most prominent atmospheric environmental problems, posing a serious threat to both the environment and public health. Therefore, exposure assessment of atmospheric pollutants is more than crucial. In this study, atmospheric environmental pollution was selected as a primary indicator to study the degree of exposure. According to the quality standard of atmospheric environment, the exposure target layer selects four major atmospheric pollutants (*SO*_2_、*NO*_2_、*PM*_2.5_、*PM*_10_) and the number of days when *AQI* reaches or exceeds Grade II. These indicators help us assess the level of pollution in the atmospheric environment. It has a positive effect on the vulnerability enhancement. The higher the exposure, the more significant the disruption to the atmospheric environment and the more vulnerable.

As greenhouse gas concentrations continue to rise, renewable surface and groundwater resources will decrease profoundly in many regions. At the same time, factors such as population growth, economic development and urbanization, the atmospheric pressure at the regional scale, especially in the coupled urban system, will witness tremendous increase in the coming decades. Sensitivity explains the degree of change when the region is subjected to natural and manufactured stress, which reflects the ease and likelihood of occurrence of atmospheric environmental problems. The evaluation indicators include diverse factors, including average annual temperature, precipitation, natural population growth rate, population density, urban built-up area, and energy consumption per unit of GDP. Among these, temperature and precipitation are essential climate characteristics for the reason that they are capable of reflecting the degree of change in the regional atmospheric environment. What is more, the natural population growth rate and population density shed light on the pressure of the population on the atmospheric environment, when the pressure exceeds the environmental carrying capacity, it can undermine the self-regulatory capacity of the natural ecosystem. Urban built-up area and energy consumption per unit of GDP reflect how urbanization and industry impact the vulnerability of the environment. Generally, the more pressure from these factors, the more sensitive the environment becomes, making it more vulnerable.

Atmospheric risks can have an impact on natural ecosystems and human social development. What is more, socio-economic pathways, scientific and technological innovation, and related governance will in turn influence the risks in the atmosphere. Risks from climate change, human societies can take adaptive actions to mitigate risks, thereby mitigating vulnerability to atmospheric changes [35]. Therefore, adaptability was chosen to evaluate atmospheric vulnerability. To be more specific, adaptability refers how well the atmosphere can bounce back to a healthy state after being damaged by external factors through its own regulation and human regulation. The adaptive capacity is mainly manifested by the active participation, and specific measures to help it recover and deliver increased recuperative capabilities., Hence, the proportion of tertiary industry to GDP, the proportion of science and technology investment to GDP, the public green area per capita and the forest coverage rate are chosen as evaluation indicators. When adaptive capacity is stronger, it means the environment can bounce back better after problems, and it becomes less vulnerable.

### Weights determination

The objective entropy weighting method was applied to determine the weights of each factor to avoid a bias of results due to subjective judgment. The study adopts the extreme difference standardization method to standardize the raw data, and the standard’s value range is 0 to 1. The specific formula is shown as follows:

(1) Standardizing the data

$$Positive\;indicators:\;x_{ij}^{\prime }=\frac{x_{ij}-\min\left(x_{1j},\;x_{2j},\;\ldots ,\;x_{mj}\right)}{\max\left(x_{1j},\;x_{2j},\;\ldots ,\;x_{mj}\right)-min\left(x_{1j},\;x_{2j},\;\ldots ,\;x_{mj}\right)}$$ (1)

$$Negative\;indicator:\;x_{ij}^{\prime }=\frac{\max\left(x_{1j},\;x_{2j},\;\ldots ,\;x_{mj}\right)-x_{ij}}{\max\left(x_{1j},\;x_{2j},\;\ldots ,\;x_{mj}\right)-min\left(x_{1j},\;x_{2j},\;\ldots ,\;x_{mj}\right)}$$ (2)

where *X*_*ij*_ indicates the *j* th index of the *i* th city

(2) Calculate the ratio *y*_*ij*_ of the *i* th city to the *j* th index

$$y_{ij}=\frac{{X_{ij}}^{*}}{\sum_{i=1}^{n}{X_{ij}}^{*}}$$ (3)

(3) Calculate the entropy value *e*_*j*_ of the *j*th index

$$e_{j}=-k\sum_{i=1}^{m}\sum_{j=1}^{p}\left(y_{ij}\times lny_{ij}\right),\;k=\frac{1}{lpm},\;0\leq e\leq 1$$ (4)

(4) Calculate the weight *w*_*j*_ of the *j* th index

$$w_{j}=(1-e_{j})/\sum_{j=1}^{n}\left(1-e_{j}\right)$$ (5)

### Calculation and classification of atmospheric vulnerability index (*AVI*)

According to the index weights determined by the entropy method, the Exposure Index (*EI*), Sensitivity Index (*SI*), and Adaptive Capacity Index (*ACI*) are calculated, respectively. Then the Atmosphere Vulnerability Index (*AVI*) is calculated according to the VSD model, the calculation formula was expressed as follows:

$$EI=\sum_{j=1}^{5}y_{ij}\omega_{j}$$ (6)

$$SI=\sum_{j=6}^{6}y_{ij}\omega_{j}$$ (7)

$$ACI=\sum_{j=12}^{4}y_{ij}\omega_{j}$$ (8)

AVI=EI+SI−ACI (9)

To be able to visually compare the spatial and temporal patterns of atmospheric environmental vulnerability, the *AVI* was classified and graded based on previous research literature [20, 24] and the characteristics of the PRD (Table 2).

**Table 2:** *AVI* classification.

level	lower	low	moderately	high	higher
range	0~0.2	0.2~0.4	0.4~0.6	0.6~0.8	0.8~1.0

### Geodetector

The Geodetector is a new statistical method for detecting the spatial variability and revealing the driving factors behind it, which is widely used in natural and social science research [36]. The Geodetector includes four modules: single factor detector, interaction detector, risk detector, and ecological detector. Factor detectors and interaction detectors were selected separately to reveal the relationship and the influence strength of indicator factors on atmospheric environment vulnerability of the PRD.

(1) Factor detector: *q* is used to measure the influence of the detector on the spatial variation of regional vulnerability. *q* is larger when the variation of the dependent variable *Y* within the study space is significantly influenced by the independent variable *X*. The expression is:

$$q=\frac{\sum_{h=1}^{L}N_{h}\sigma_{h}^{2}}{N\sigma^{2}}$$ (10)

*h* = 1,……; *L* is the layering of detection factor *X*; *N*_*h*_、*N* is the he number of units representing layer *h* and the entire area, respectively; $\sigma_{h}^{2}$、 *σ*^2^ is the variance of the atmospheric vulnerability of layer h and the entire region, respectively; *q*∈[0,1],The larger the *q*, the stronger the explanatory power of *X*_*i*_ on the spatial differentiation of atmospheric environmental vulnerability.

(2) Interaction detection: It is used to identify the explanation degree of the interaction between different factors on the dependent variable *Y*. The types of interactions between the two factors are shown in Table 3.

**Table 3:** Interaction types.

Judgment basis	Interaction
q(X1∩X2)<Min(q(X1),q(X2))	Nonlinear attenuation
Min(q(X1),q(X2))<q(X1∩X2)<Max(q(X1),q(X2))	Single-factor nonlinear weakening
q(X1∩X2)>Max(q(X1),q(X2))	Two-factor enhancement
q(X1∩X2) = q(X1)+q(X2)	Independent
q(X1∩X2)>q(X1)+q(X2)	Nonlinear enhancement

## Study area and data sources

### Study area

**Fig 2:**
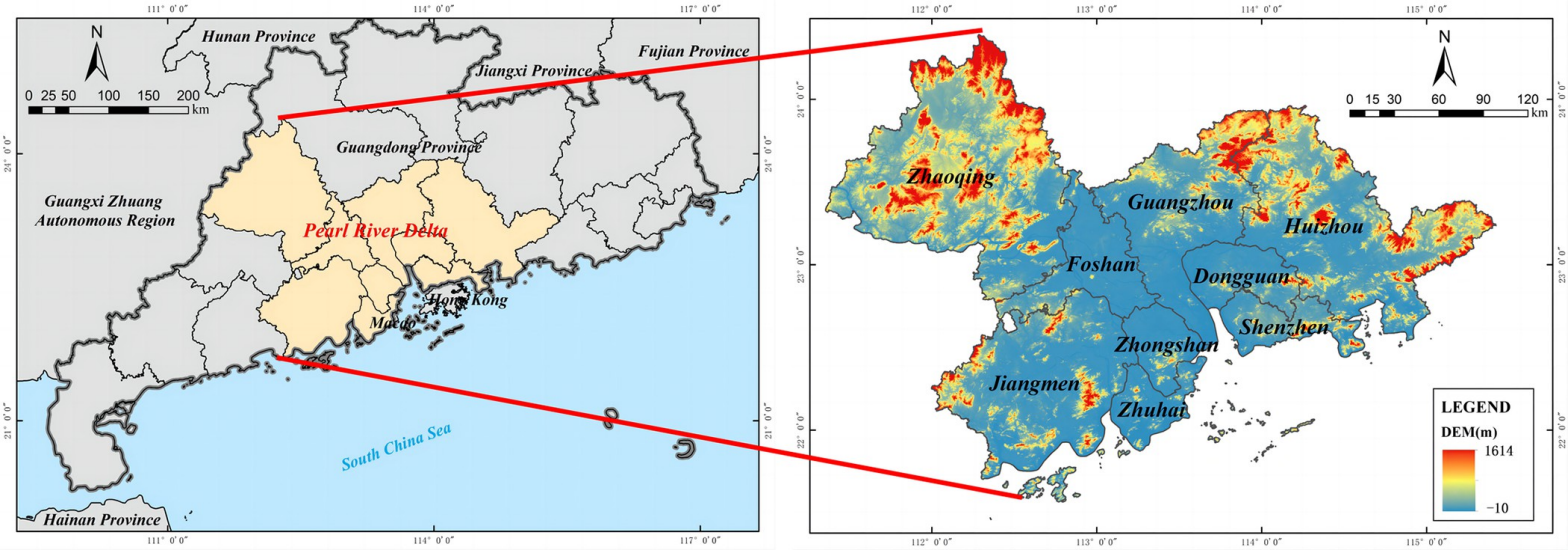
Study area. Reprinted background map from the National Catalogue Service for Geographic Information (www.webmap.cn) under a CC BY license, with permission from the Ministry of Natural Resources of China, original copyright 2020.

The PRD is located in South China, including nine cities: Guangzhou, Shenzhen, Zhuhai, Foshan, Huizhou, Dongguan, Zhongshan, Jiangmen, and Zhaoqing (Fig 2). It covers a regional area of about 54 770.21 km^2^, and stands as one of China’s three major urban agglomeration and economic centers. It is a pioneer area of China’s reform and opening and an essential economic growth pole in South China. As of 2020, the region’s total population reached 78 235 400, accounting for 61.97% of the total population of Guangdong Province, with a population density of about 0.23 million people per square kilometer. The PRD’s economy has experienced rapid development, becoming one of the fastest growing and the most vibrant regions in China. The GDP of the PRD reached 89 523.93 hundred million yuan in 2020, an increase of 31.27% compared with 2016. The proportion of the tertiary industry, primarily comprising the financial and service sectors, has generally shown an increasing trend. Along with rapid economic development, the PRD has also suffered from a more serious ecological environment. The *AQI* in 2020 ranges from 88.0% to 97.8%, with an average of 92.9%, lower than the provincial average (95.5%).

### Data sources

The scope of administrative boundary of the PRD was sourced from the Data Center for Resource and Environmental Sciences of the Chinese Academy of Sciences (http://www.resdc.cn/). The digital elevation model (DEM) of the PRD was acquired from the Geospatial Data Cloud (https://www.gscloud.cn/). Meteorological data, which includes *SO*_2_ concentration, *NO*_2_ concentration and *PM*_2.5_ concentration, *PM*_10_ concentration, *AQI*, temperature and precipitation, etc. were collected from the environmental quality bulletins released by the ecological environment bureaus of each city. Socio-economic data were extracted from the statistical yearbooks released by each city, including natural population growth rate, energy consumption per unit of GDP, the proportion of tertiary industry in GDP and the balance of investment in science and technology in GDP. In addition, population density, urban built-up area, green area per capita, and forest coverage rate were obtained from the statistical yearbook of urban construction issued by the Ministry of Housing and Urban-Rural Development.

## Results

### Exposure index

**Fig 3:**
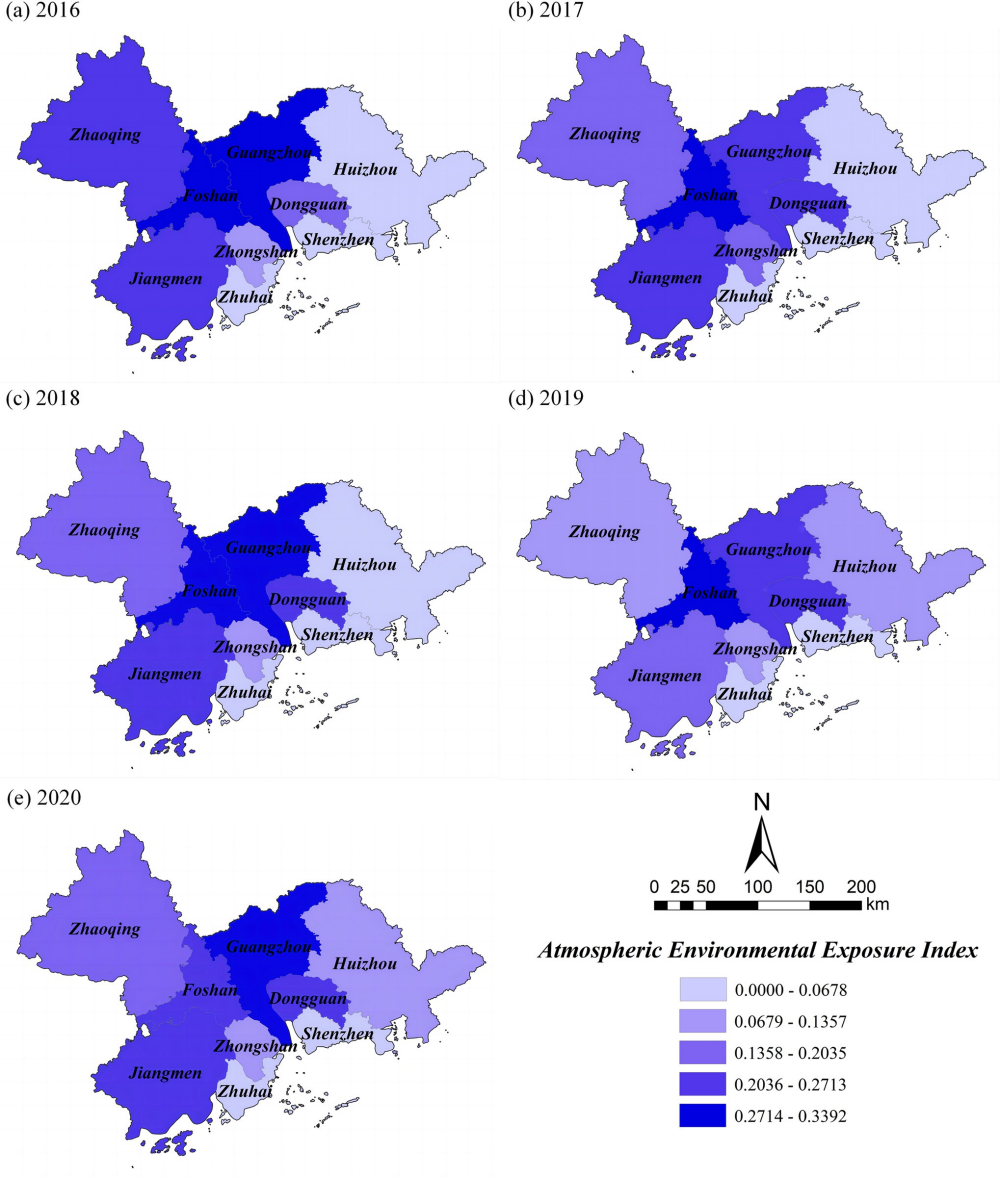
Spatial distribution of *EI* in the PRD. Reprinted background map from the National Catalogue Service for Geographic Information (www.webmap.cn) under a CC BY license, with permission from the Ministry of Natural Resources of China, original copyright 2020.

Through the calculation of the factors, the *EI* of the atmospheric environment ranged from 0 to 0.3392 from 2016 to 2020, and the five-year average values of *EI* showed a slight increase of 2.34% overall (Table 4). In terms of spatial distribution, there is a pattern of "high in the middle and low on both sides" (Fig 3), and the regions with high exposure index are mainly concentrated in Guangzhou and Foshan in the central PRD, followed by Dongguan, Zhongshan, Jiangmen, and Zhaoqing. Among them, the annual average concentration of *SO*_2_ in Guangzhou and the concentration of *PM*_2.5_ and *PM*_10_ in Foshan are higher. There are more types and contents of air pollutants, so the exposure to the air environment system is higher. The regions with lower exposure index are mainly distributed in Huizhou, Shenzhen, these areas are coastal cities with fast air movement and low levels of pollutants, resulting in better air quality relative to inland cities.

**Table 4:** *EI* of the PRD from 2016 to 2020.

City	2016	2017	2018	2019	2020
Guangzhou	0.2796	0.2448	0.3036	0.2524	0.2911
Shenzhen	0.0411	0.0377	0.0208	0.0308	0.0598
Zhuhai	0.0489	0.0599	0.0457	0.0517	0.0525
Foshan	0.2875	0.2888	0.3392	0.2885	0.2512
Huizhou	0.0000	0.0225	0.0277	0.0967	0.1169
Dongguan	0.1950	0.2077	0.2509	0.2704	0.2528
Zhongshan	0.1350	0.1738	0.1346	0.1283	0.1037
Jiangmen	0.2448	0.2540	0.2329	0.1778	0.2142
Zhaoqing	0.2298	0.1580	0.1936	0.1048	0.1535

### Sensitivity index

**Fig 4:**
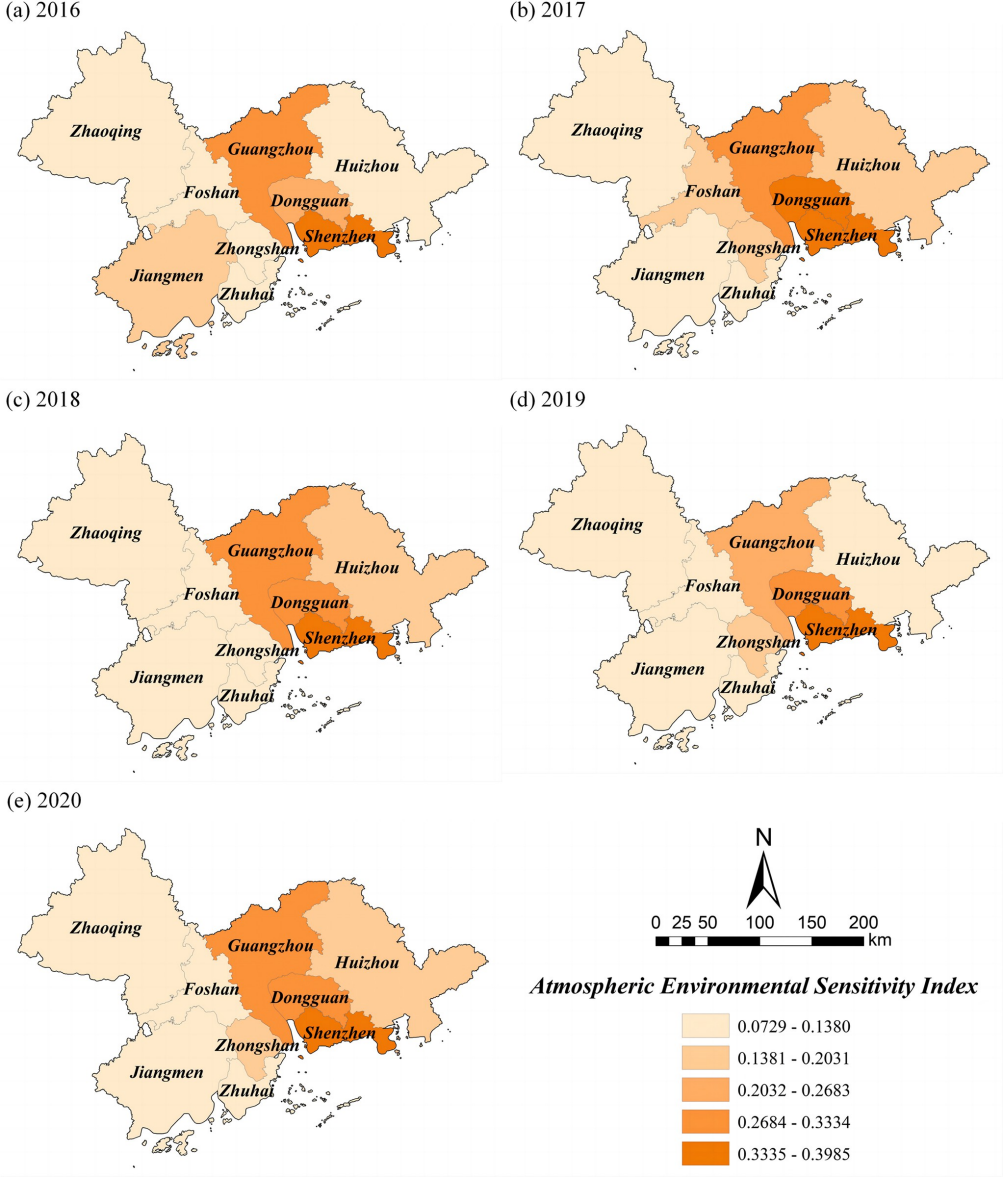
Spatial distribution of *SI* in the PRD. Reprinted background map from the National Catalogue Service for Geographic Information (www.webmap.cn) under a CC BY license, with permission from the Ministry of Natural Resources of China, original copyright 2020.

In contrast to the temporal changes in exposure, sensitivity showed a fluctuating increase and a higher increase of 3.77%. As can be seen from Table 5, the *SI* of the PRD ranged from 0.0729 to 0.3985, and the average values of *SI* in these five years were 0.1829 (2016), 0.2018 (2017), 0.1857 (2018), 0.1868 (2019), and 0.1898 (2020). The spatial distribution shows a pattern of "high in the middle and low on both sides" (Fig 4). Most areas with high *SI* are concentrated in Guangzhou, Dongguan, and Shenzhen, where the population is concentrated, the building area is large, and the urbanization level is high. The *SI* of Huizhou in the eastern part of the PRD and Zhaoqing, Foshan and Zhuhai in the western part of the PRD are relatively low, because the population density in these areas is low, and the urban expansion rate is relatively reasonable.

**Table 5:** *SI* of the PRD from 2016 to 2020.

City	2016	2017	2018	2019	2020
Guangzhou	0.2793	0.3039	0.2994	0.2485	0.2735
Shenzhen	0.3985	0.3471	0.3485	0.3434	0.3377
Zhuhai	0.1316	0.1317	0.1166	0.1280	0.1209
Foshan	0.1258	0.1644	0.1271	0.1307	0.1315
Huizhou	0.1277	0.1411	0.1713	0.1373	0.1575
Dongguan	0.2669	0.3533	0.3224	0.3327	0.2918
Zhongshan	0.0972	0.1661	0.1306	0.1722	0.1903
Jiangmen	0.1458	0.0930	0.0770	0.0729	0.1090
Zhaoqing	0.0731	0.1154	0.0786	0.1157	0.0958

### Adaptive capacity index

**Fig 5:**
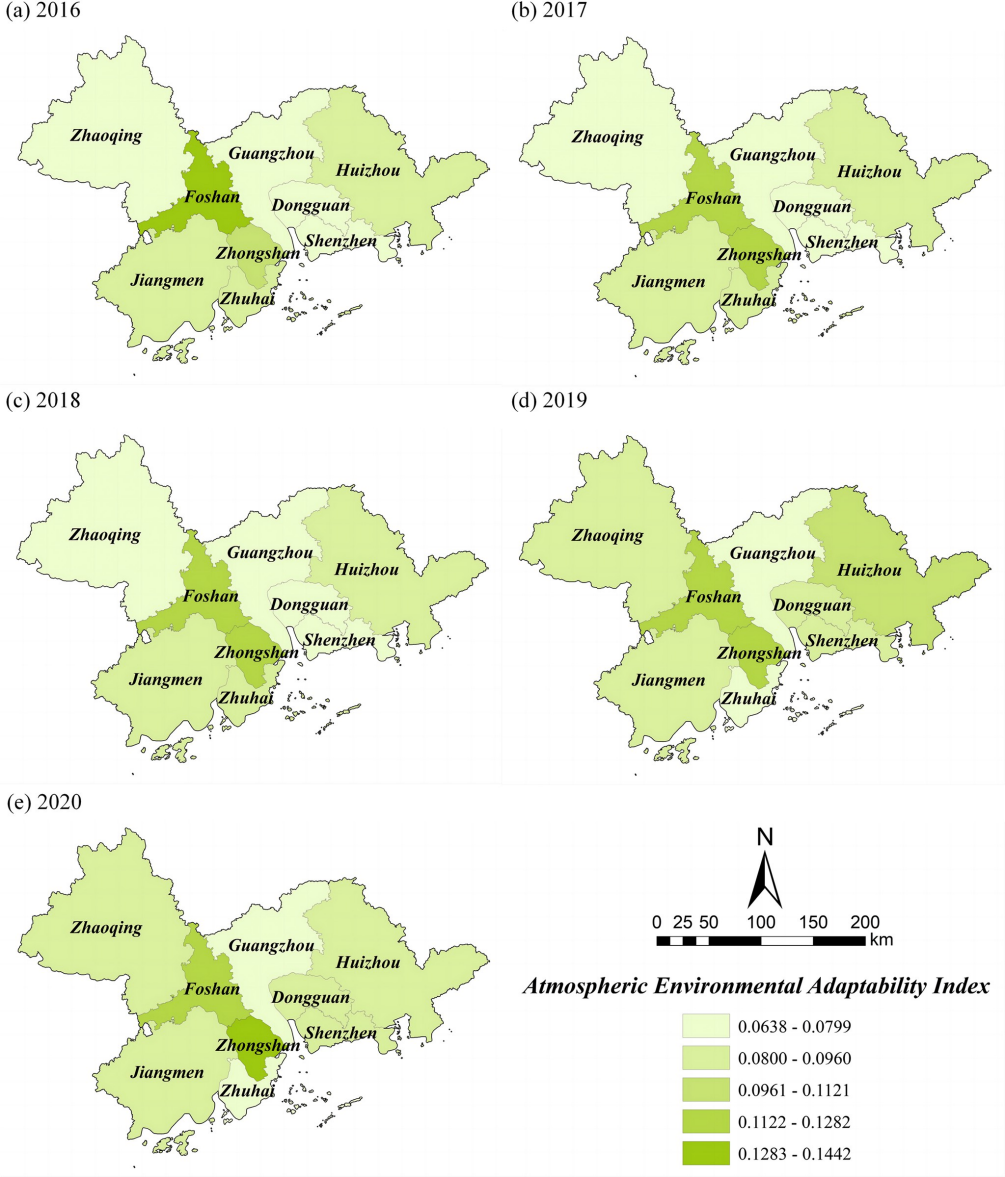
Spatial distribution of *ACI* in the PRD. Reprinted background map from the National Catalogue Service for Geographic Information (www.webmap.cn) under a CC BY license, with permission from the Ministry of Natural Resources of China, original copyright 2020.

There is a negative correlation between adaptability and atmospheric environmental vulnerability. The *ACI* of the PRD from 2016 to 2020 ranged from 0.0638 to 0.1442 (Table 6). The mean values of the *ACI* for these five years were 0.0841 (2016), 0.0896 (2017), 0.0865 (2018), 0.0900 (2019), and 0.0925 (2020), and showed a significant upward trend overall, with an increase of 9.99%, it also shows that the PRD has made significant improvements in ecological protection and optimization of industrial structure. From Fig 5, the spatial distribution shows a pattern of "low in the middle and high on both sides". The high adaptable areas are mainly Foshan, Zhongshan, and Huizhou, followed by Jiangmen, Zhaoqing, and Zhuhai. In contrast, the areas with low adaptability are primarily concentrated in Guangzhou, Dongguan and Shenzhen in the central PRD, which are influenced by the per capita green area and forest cover, with high vegetation cover and better ecological environment on the east and west sides of the PRD, so the resistance of the atmospheric environment system is more robust. However, the *ACI* in the central PRD is gradually strengthening, indicating that the vegetation greening and environmental protection are also increasing with the socio-economic development of Guangzhou, Dongguan and Shenzhen.

**Table 6:** *ACI* of the PRD from 2016 to 2020.

City	2016	2017	2018	2019	2020
Guangzhou	0.0676	0.0695	0.0657	0.0659	0.0745
Shenzhen	0.0638	0.0710	0.0689	0.0857	0.0871
Zhuhai	0.0807	0.0862	0.0804	0.0669	0.0681
Foshan	0.1288	0.1253	0.1165	0.1212	0.1156
Huizhou	0.0805	0.0908	0.0860	0.0960	0.0938
Dongguan	0.0696	0.0749	0.0744	0.0886	0.0867
Zhongshan	0.1049	0.1171	0.1183	0.1258	0.1442
Jiangmen	0.0887	0.0926	0.0899	0.0801	0.0819
Zhaoqing	0.0727	0.0791	0.0780	0.0802	0.0806

### Atmospheric environmental vulnerability

**Fig 6:**
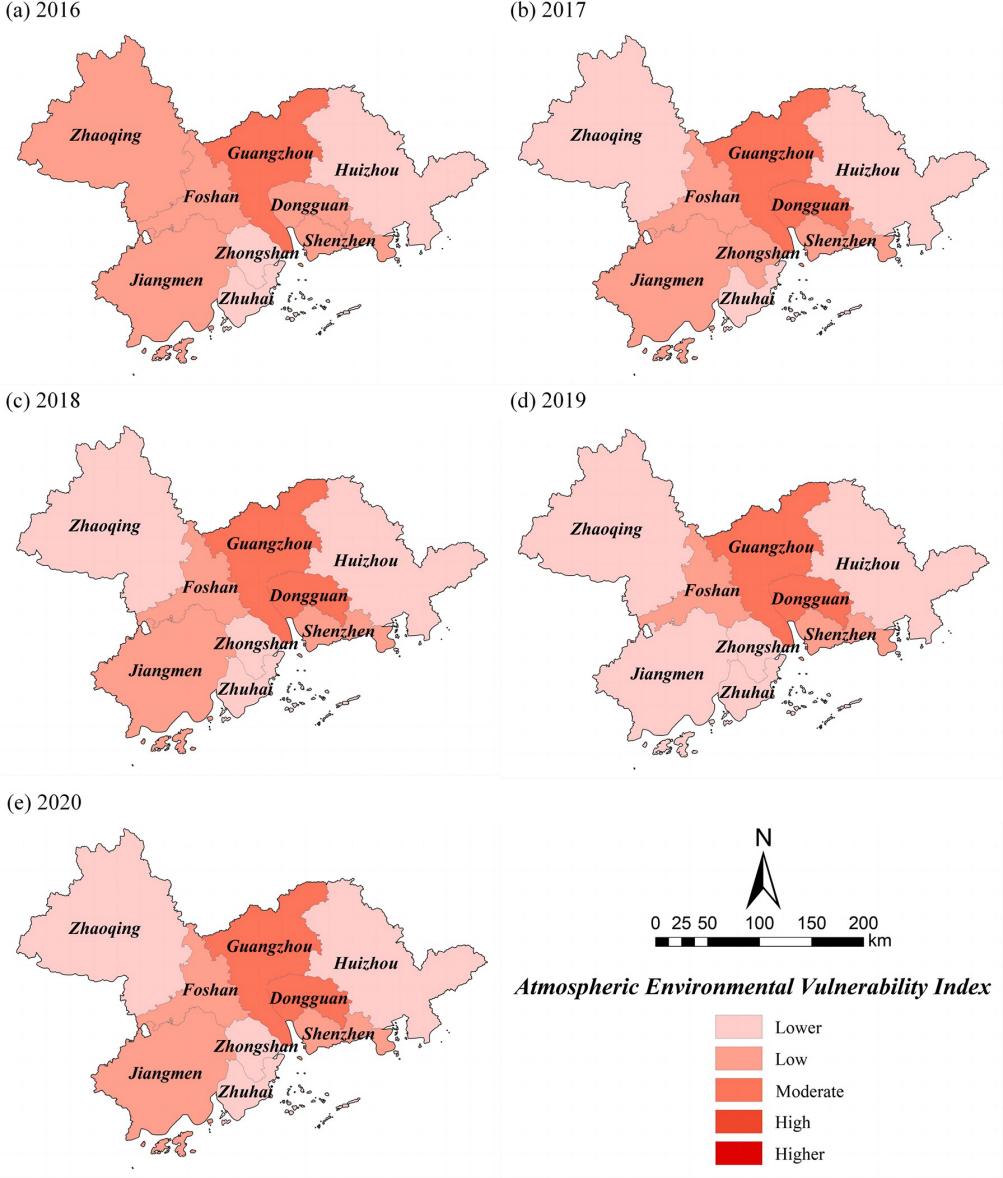
*AVI* of each city from 2016 to 2020.

During the 2016–2020 period, the atmospheric environmental vulnerability of the PRD shows large municipal differences (Fig 6). The upward trend is in Huizhou and Dongguan, with Dongguan showing a fluctuating upward trend; the areas showing a fluctuating downward trend are Guangzhou, Shenzhen, Jiangmen and Zhaoqing; the atmospheric environmental vulnerability of Foshan first rises and then declines; while that of Zhuhai maintains a relatively low and stable level of vulnerability; and the atmospheric environmental vulnerability that fluctuates considerably is that of Zhongshan, but is generally low vulnerable.

**Fig 7:**
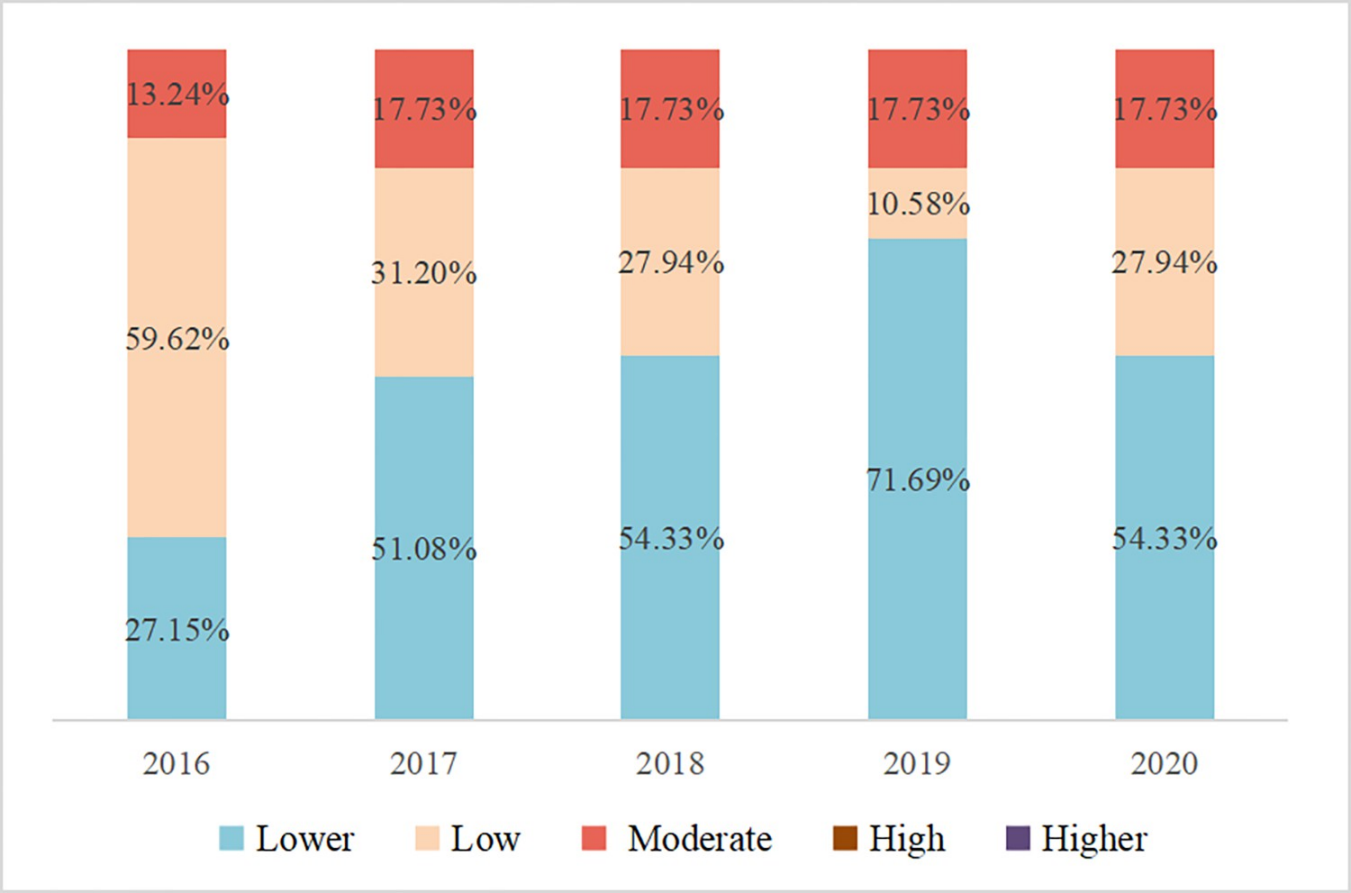
Multi-year average values of *AVI* for each city in the PRD.

From the Fig 7, it can be seen that the average values of vulnerability in Zhuhai, Huizhou, Zhongshan, and Zhaoqing are lower, with the average multi-year vulnerability values of 0.1010, 0.1103, 0.1643, and 0.1855, respectively, which are all below 0.2. The *AVI* of Shenzhen, Foshan and Jiangmen are between 0.2 and 0.4, indicating that the overall atmospheric environment is in a low vulnerability state. The two regions with the highest *AVI* are Guangzhou and Dongguan, with 0.4866 and 0.700, indicating that the overall atmospheric environment vulnerability is in a moderate state.

**Fig 8:**
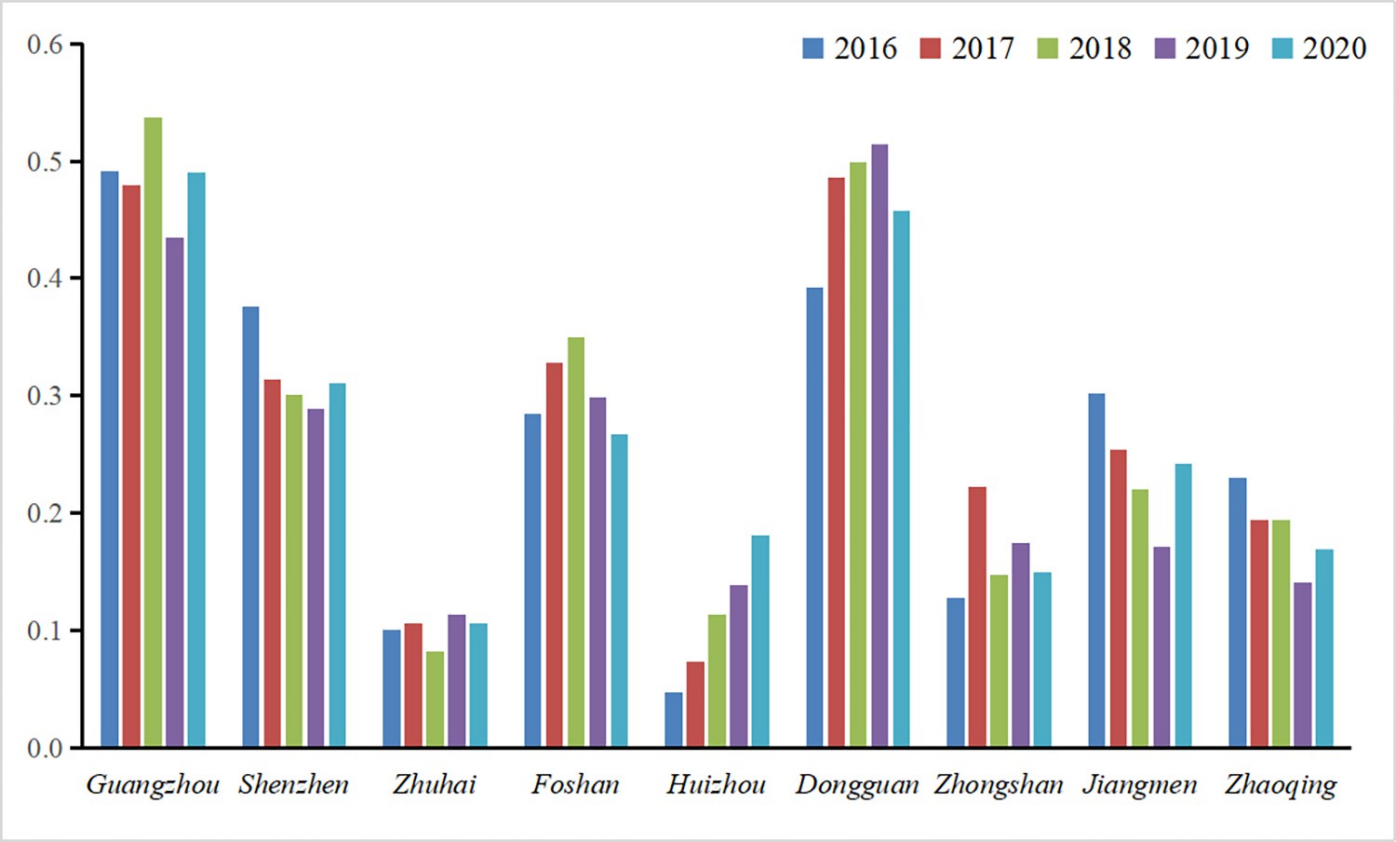
Spatial distribution of *AVI* in the PRD. Reprinted background map from the National Catalogue Service for Geographic Information (www.webmap.cn) under a CC BY license, with permission from the Ministry of Natural Resources of China, original copyright 2020.

**Fig 9:**
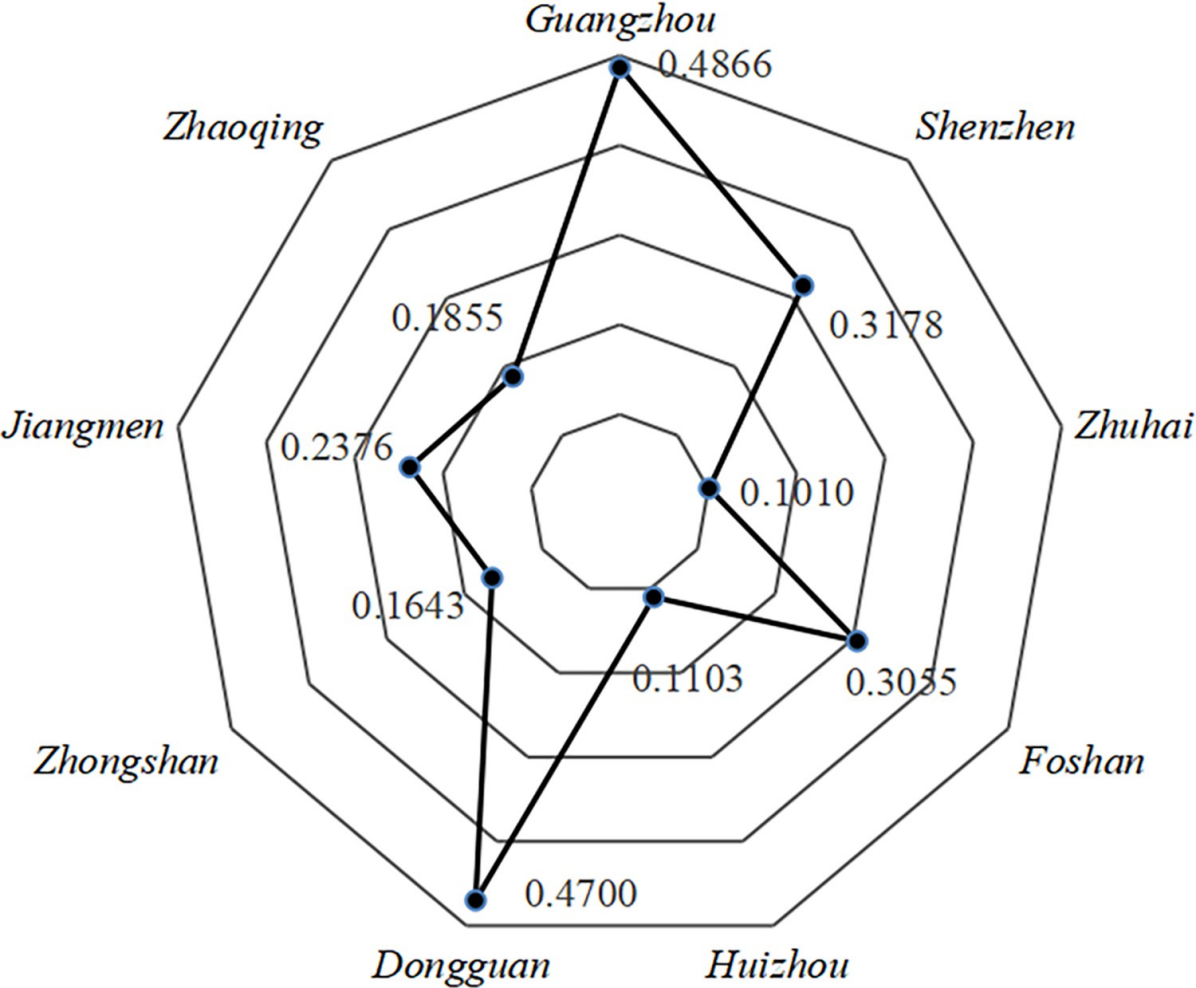
Proportion of *AVI* from 2016 to 2020 in the PRD.

The spatial visualization was carried out by GIS (Fig 8), and the percentage of each atmospheric environmental vulnerability grade in different years was counted (Fig 9). As can be seen from Fig 8, the areas with lower atmospheric vulnerability are mainly located in Huizhou in the eastern part of the PRD, and Zhongshan and Zhuhai in the southern part. The distribution of regions with higher atmospheric vulnerability in different periods varies greatly, especially in the western PRD, where the atmospheric vulnerability of Zhaoqing declined from low vulnerability in 2016 to lower vulnerability in 2017 and has been stable at a low vulnerability since then; the atmospheric vulnerability of Jiangmen was at lower level in all other periods except 2019 when it was at low vulnerability level; And the atmospheric environmental vulnerability level of Guangzhou, Dongguan, Foshan, and Shenzhen in the central PRD has been located at a moderately level.

From Fig 9, the proportion of the low vulnerable grade area was the largest in 2016, accounting for 59.62%, followed by the lower vulnerable grade area, accounting for 27.15%, and the most minor proportion of the moderately vulnerable grade area, accounting for only 13.24%. the lower vulnerable grade area expanded in 2017, accounting for 51.08%, the low vulnerable grade area shrank, accounting for 31.20%, while the proportion of moderately vulnerable grade area began to increase, reaching 17.73%.the area of lower vulnerable grade further expanded, accounting for 54.33% in 2018, the area of low vulnerable quality further shrank, accounting for 27.94%, and the area of moderately vulnerable grade remained unchanged, accounting for 17.73%; in 2019, the proportion of the lower vulnerable grade area is the largest among the five years, reaching 71.69%, while the proportion of the low vulnerable grade is the smallest among the five years, accounting for only 10.58%, and the area of the moderately vulnerable grade remains unchanged. Compared with the previous year, the area of lower vulnerable grade shrinks in 2020, with the proportion falling back to 54.33%, while the area of low vulnerable grade increases, with the proportion rising to 27.94%, and the area of moderately vulnerable grade remains unchanged, with the proportion stable at 17.73%.

In conclusion, the proportion of lower vulnerable class areas of the atmospheric environment in the PRD from 2016 to 2020 shows a significant trend of increasing. The proportion of low vulnerable class areas gradually compresses, while the moderately vulnerable class area first increases from 13.24% to 17.73% and then remains unchanged. None of the atmospheric vulnerability classes in the study area are high and higher from 2016 to 2020.

## Influencing factors of *AVI*

The atmospheric environmental vulnerability of the PRD showed differentiated spatial distribution characteristics, and the Geodetector were used to explore its main influencing factors. The dependent variable is set as the *AVI*, and the independent variables are set as the evaluation factors. The analysis was carried out from two aspects, firstly, to analyze the influence of each index on the spatial heterogeneity of atmospheric environmental vulnerability in the PRD, and on the other hand, to analyze whether their interaction enhances the influence of atmospheric environmental vulnerability.

### Factor detection of *AVI*

As shown in Table 7, the top five factors of the explanatory power of the indicator factors on the spatial variability of the atmospheric environmental vulnerability of the PRD were urban built-up area, forest cover, green area per capita, population density and the proportion of tertiary industry in GDP in 2016. In 2017, the top five factors are urban built-up area, population density, forest cover, *NO*_2_ concentration and annual average temperature, and their *q* values are 0.7312, 0.5956, 0.5679, 0.5509 and 0.4844, respectively. In 2018, the top five factors of the *AVI* are urban built-up area, number of days when *AQI* reaches or exceeds Grade II, *SO*_2_ concentration, *PM*_2.5_ concentration and *PM*_10_ concentration. From large to small, urban built-up area, population density, *PM*_2.5_ concentration, *SO*_2_ concentration and *NO*_2_ concentration are the top five factors in 2019. The top five factors in 2020 are urban built-up area, annual average temperature, the proportion of tertiary industry in GDP, *PM*_2.5_ concentration and population density, and their q-values are 0.7519, 0.4634, 0.4555, 0.4074 and 0.4009, respectively.

**Table 7:** Factor detectors of *AVI* in the PRD.

Factor layer	*q*						
	2016	2017	2018	2019	2020年	average	ranking
***SO*_2_** Concentration	0.2308	0.3919	0.5186	0.3811	0.2156	0.3476	8
***NO*_2_** Concentration	0.3527	0.5509	0.4167	0.3477	0.2939	0.3924	6
***PM*_2.5_** Concentration	0.4067	0.3243	0.5151	0.3938	0.4074	0.4095	3
***PM*_10_** Concentration	0.4168	0.0895	0.5000	0.2297	0.2156	0.2903	10
Number of days when AQI reaches or exceeds Grade II	0.2845	0.4702	0.6118	0.3240	0.0615	0.3504	7
Annual average temperature	0.1193	0.4844	0.4596	0.0593	0.4634	0.3172	9
Average annual precipitation	0.4732	0.1370	0.3117	0.0970	0.3123	0.2662	12
Natural population growth rate	0.0956	0.3546	0.3715	0.0509	0.0676	0.1880	13
Population density	0.5202	0.5956	0.4929	0.6258	0.4009	0.5271	2
Urban built-up area	0.7664	0.7312	0.6645	0.7158	0.7519	0.7260	1
Energy consumption per GDP	0.1406	0.2736	0.1458	0.1019	0.2747	0.1873	14
Proportion of tertiary industry in GDP	0.5168	0.2895	0.4158	0.3391	0.4555	0.4033	4
Proportion of science and technology investment in GDP	0.0857	0.0475	0.1219	0.2269	0.0790	0.1122	15
Public green area per capita	0.6371	0.4083	0.0064	0.2170	0.0945	0.2726	11
Forest coverage	0.6888	0.5679	0.2082	0.2428	0.2634	0.3942	5

Although the magnitude of the explanatory power of the 15 evaluation factors varies among years, the average of 5-year q-values indicates that the explanatory power of the *AVI* is generally as follows: urban built-up area (0.7260) > population density (0.5271) > *PM*_2.5_ concentration (0.4095) > proportions of tertiary industry in GDP (0.4033) > forest cover (0.3942) > *NO*_2_ concentration (0.3924) >number of days when *AQI* reaches or exceeds Grade II (0.3504)> *SO*_2_ concentration (0.3476)>annual average temperature (0.3172)> *PM*_10_ concentration (0.2903)>area of public green space per capita (0.2726)>annual average precipitation (0.2662) > natural population growth rate (0.1880) > energy consumption per unit of GDP (0.1873) > proportion of science and technology investment in GDP (0.1873).

Combining the higher top five factors and the multi-year *q* values from 2016–2020, it is clear that urban built-up area and population density are the main influencing factors affecting the change of *AVI* in the PRD, especially the urban built-up area, which has been the highest *q* value and the most influential in the five years. *PM*_2.5_ concentration, the proportion of tertiary industry in GDP, forest coverage, *NO*_2_ concentration, the number of days with *AQI* at or above Grade II, *SO*_2_ concentration and annual average temperature are important influencing factors of *AVI*. Other indicators such as *PM*_10_ concentration, per capita public green space, average annual precipitation, natural population growth rate, energy consumption per unit of GDP, proportion of science and technology investment in GDP explain the *AVI* to a lesser extent.

### Interaction detection of *AVI*

**Fig 10:**
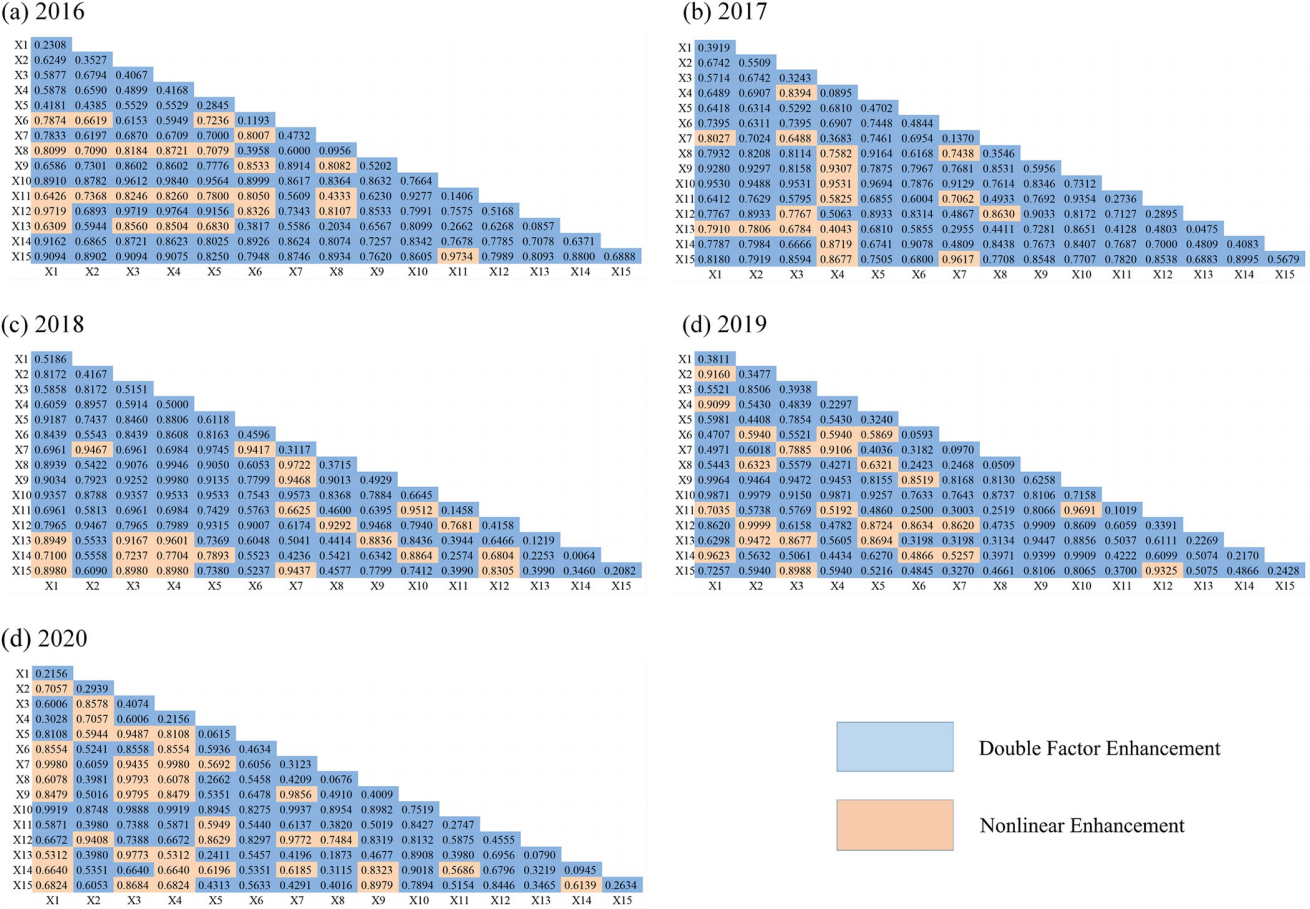
Interaction detection of *AVI* in the PRD.

The joint effect of multiple factors on atmospheric environmental vulnerability may differ from the effect of individual factors. The interaction detection of 15 evaluation index factors of atmospheric environmental vulnerability in the PRD in 2016–2020 using the interaction detector produced 105 interaction results (Fig 10), which all showed enhancement, mainly divided into two types of double factor enhancement and non-linear enhancement, which indicated that the interaction between any two factors had a more significant effect on regional atmospheric environmental. This suggests that the effect of interaction between any two factors on regional atmospheric vulnerability is more obvious than the effect of a single factor.

The top five sets of interactive factors with the most potent explanatory power for the *AVI* of the PRD in 2016 are urban built-up area ∩ *PM*_10_ concentration (0.9840), followed by the proportion of tertiary industry to GDP ∩ *PM*_10_ concentration (0.9764), forest cover ∩ energy consumption per GDP (0.9734), the proportion of tertiary industry to GDP ∩ *SO*_2_ concentration (0.9719) and *PM*_2.5_ concentration ∩ the proportion of tertiary industry to GDP (0.9719).

In 2017, urban built-up area ∩ Number of days when *AQI* reaches or exceeds Grade II (0.9694), forest cover ∩ average annual precipitation (0.9617), urban built-up area ∩ *PM*_2.5_ concentration (0.9531), urban built-up area ∩ *PM*_10_ concentration (0.9531), and urban built-up area ∩ *SO*_2_concentration (0.9530), these five sets of interactions have a strong impact on the vulnerability of the atmospheric environment in the PRD.

The top five interacting factors with the strongest explanatory on the *AVI* in 2018 are population density ∩ *PM*_10_ concentration (0.9980), *PM*_10_ concentration ∩ natural population growth rate (0.9946), Number of days when *AQI* reaches or exceeds Grade II ∩ average annual precipitation (0.9745), average annual precipitation ∩ natural population growth rate (0.9722), and average annual precipitation ∩ natural population growth rate (0.9723), *PM*_10_ concentration ∩ Proportion of science and technology investment in GDP (0.9601).

The effects of factor interactions changed considerably over time.The top five sets of interactive factors with the most substantial explanatory power for the *AVI* in 2019 are *NO*_2_ concentration ∩ share of tertiary industry in GDP (0.9999), followed by *NO*_2_ concentration ∩ urban built-up area (0.9979), *SO*_2_ concentration ∩ population density (0.9964), population density ∩ the proportion of tertiary industry to GDP (0.9909), and urban built-up area ∩ public green space per capita (0.9909) in 2019. The interactions of *NO*_2_ concentration, tertiary industry in GDP, urban built-up area, per capita public green area, *SO*_2_ concentration and population density in 2019 have a strong impact on the *AVI*.

The top five interacting factors with the strongest explanatory on the *AVI* in 2020 are *SO*_2_ concentration ∩ average annual precipitation (0.9980), *PM*_10_ concentration ∩ average annual precipitation (0.9980), urban built-up area ∩ average annual precipitation (0.9937), urban built-up area ∩ *SO*_2_ concentration (0.9919), urban built-up area ∩ *SO*_2_ concentration (0.9920), and urban built-up area∩*PM*_10_ concentration (0.9919).

The combined results of factor detection and interaction detection show that urban built-up area, *PM*_2.5_ concentration, *SO*_2_ concentration, population density and the proportion of tertiary industry to GDP are the critical drivers of atmospheric vulnerability in the PRD. These influencing factors will also become the main content and direction affecting atmospheric environmental protection and planning, and atmospheric environmental management in the PRD. From another aspect, the appropriate scale of urban development, rationalization of industrial structure, and legalization of air pollution prevention and control will become the main objectives of air environment management in the PRD in the future.

## Conclusion and discussion

### Conclusion

1. Between 2016 to 2020, the *SI* and *ACI* of the PRD exhibit a fluctuating upward trend. The *EI* shows a pattern of increase, decline, then increase again, with large year-to-year fluctuations. What shall be noticed is that, the rise of the adaptability index significantly weakens the atmospheric environmental vulnerability in the PRD.
2. In general, the *AVI* the PRD is mainly in the lower and low vulnerability categories. These two categories together make up more than 83.17% on average over the years, with the highest reaching 86.76% (2016). On the other hand, the proportion of the area of the moderate vulnerability class is less, and no high or higher vulnerability occurs. The atmospheric environment vulnerability index of the PRD in 2016–2020 is 0.2611, 0.2730, 0.2714, 0.2525, and 0.2635, showing a fluctuating trend. Still, the average value of the multi-year vulnerability index stands at 0.2643, which is mildly vulnerable.
3. From the perspective of municipalities, there exist remarkable differences in the *AVI* among the nine cities in the PRD. The cities of Guangzhou, Dongguan, Foshan, and Shenzhen, particularly Guangzhou and Dongguan, tend to have moderate vulnerability levels over the years. These areas are densely populated and witness frequent socio-economic activities, which are prone to air pollution problems. The lower vulnerability is mainly distributed in the areas with the good natural environment and less human interference on the eastern and western sides of the PRD, such as Huizhou, Zhaoqing and Zhuhai. In addition, Zhongshan evolved from slightly vulnerable in 2016 to mildly vulnerable in 2017, then back to mildly vulnerable in 2018, where it remained stable. The vulnerability index first increased and then decreased. Also, Jiangmen transformed from mildly vulnerable to mildly vulnerable in 2019, and then converted to mildly vulnerable in 2020, with the vulnerability index reducing and then increasing.
4. The Geodetector findings show that compared with the role of individual factors, the *AVI* of the PRD is more susceptible to interactions among factors. It represents the result of the combined effect of natural, social, and economic factors. Among these factors, key drivers include urban built-up area, *PM*_2.5_ concentration, *SO*_2_ concentration, population density and the proportion of tertiary industry in GDP. As such, when boosting the socio-economic development and urbanization of the PRD, it is imperative to consider the combined impact of these factors on the air quality and ecosystem. To put it in detail, these aspects include regional suspended delicate particulate matter and sulfur oxide emissions, urban expansion, and industrial structure. By doing so, air environmental protection and prevention, coordinated ecological protection and economic development, and high-quality development of the PRD can be delivered.

### Discussion

The PRD urban agglomeration is one of China’s most active urban agglomerations, marked by robust economic and social development. Nonetheless, the frequent human activities have exerted tremendous pressure on the atmospheric environment and ecosystem. Notably, this study is capable of offering fresh perspective and empirical analysis for the atmospheric environmental vulnerability and high-quality development of the urban agglomeration. The *AVI* of the PRD urban agglomeration from 2016 to 2020 shows a first increase followed by a decrease. which indicates that the atmospheric environmental vulnerability of the region has gradually decreased in recent years. Such improvement can be attributed to the emphasis on the prevention and control of atmospheric pollution and the construction of ecological civilization. These efforts have improved the adaptive capacity of the atmospheric environment and ecosystem to external disturbances. Guided byobjectives of the new development concept and ecological civilization ideology, the PRD has carried out the Blue-Sky Protection Campaign, precisely managed the significant problems of air pollutant emissions in critical areas, attached importance to the coordinated development of the economy and environment, and promoted the city cluster to achieve high-quality economic and social development.

However, this paper still needs to improve and solve problems. Quantitative assessment of atmospheric environmental vulnerability remains relatively understudied, and the field is still in its early stages of exploration. What is more, the relevant theories still need to be completed. The 15 evaluation indicators are selected to evaluate the vulnerability of the atmospheric environment. Although many references have been made to the literature for the selection of evaluation indicators and the calculation of weights, the factors contributing to the vulnerability of the atmospheric environment are relatively numerous and complex. Therefore, the evaluation indicator system constructed may have limitations and should be refined in future research. On the time scale, due to the difficulty of collecting relevant information and data, only five years, from 2016 to 2020, are used for the evaluation of the atmospheric environment of the PRD. Atmospheric pollution in the PRD is characterized by a significant regional dimension, with obvious interactions between cities, making it difficult to solve the air pollution problem by considering the prevention and control of air pollution in individual cities only from the perspective of administrative divisions. Therefore, regional joint prevention and control among cities is an effective means to solve regional air pollution problems. In the study area, the research scale of this paper falls in the city area. However, the natural resource endowment, socio-economic conditions, and development history of each county and district are different, which brings about certain limitations. Notably, if the research scale can be further refined, the evaluation effect will be more accurate and precise. These are the main research directions and prospects for future atmospheric vulnerability studies.
